# Marine invertebrate biodiversity from the Argentine Sea, South Western Atlantic

**DOI:** 10.3897/zookeys.791.22587

**Published:** 2018-10-22

**Authors:** Gregorio Bigatti, Javier Signorelli

**Affiliations:** 1 Laboratorio de Reproducción y Biología Integrativa de Invertebrados Marinos, (LARBIM) IBIOMAR-CONICET. Bvd. Brown 2915 (9120) Puerto Madryn, Chubut, Argentina Laboratorio de Reproducción y Biología Integrativa de Invertebrados Marinos Puerto Madryn Argentina; 2 Universidad Nacional de la Patagonia San Juan Bosco, Boulevard Brown 3051, Puerto Madryn, Chubut, Argentina Universidad Nacional de la Patagonia San Juan Bosco Puerto Madryn Argentina; 3 Facultad de Ciencias Ambientales, Universidad Espíritu Santo, Ecuador Universidad Espíritu Santo Guayaquil Ecuador

**Keywords:** Argentina, Arthropoda, checklist, Mollusca, taxonomy

## Abstract

The list of marine invertebrate biodiversity living in the southern tip of South America is compiled. In particular, the living invertebrate organisms, reported in the literature for the Argentine Sea, were checked and summarized covering more than 8,000 km of coastline and marine platform. After an exhaustive literature review, the available information of two centuries of scientific contributions is summarized. Thus, almost 3,100 valid species are currently recognized as living in the Argentine Sea. Part of this dataset was uploaded to the OBIS database, as a product of the Census of Marine Life-NaGISA project. A list of 3,064 valid species, grouped into 1,662 genera distributed in 808 families and 23 phyla, was assessed. The best represented taxa were Arthropoda and Mollusca, contributing approximately with the 50% of the mentioned species in the literature. Cumulative species curves were analyzed in order to estimate the percentage of marine invertebrate biodiversity that is currently known. However, no model fit to our data, showing that the recorded species represent less than 50% of the expected marine invertebrate biodiversity for the Argentine Sea. The great surface of the Argentine Marine Platform (6,581,500 km^2^) and the relative low effort in collecting and studying new species due to economical restrictions could explain the low fraction of described species. The training of new taxonomists, as well as, the support of projects that contribute to the knowledge of marine invertebrate biodiversity from South Western Atlantic is recommended.

## Project details

**Project title**: Marine Invertebrate Biodiversity from the Argentine Sea (South Western Atlantic).

**Personnel**: Gregorio Bigatti (data collector, data manager, project director); Javier H. Signorelli (collection identifier, data collector, data manager).

**Funding**: This project was partially supported by Census of Marine Life, Nagisa Project, SARCE, PICT 2014-640.

**Study area descriptions**: The Large Marine Ecosystems (LMEs) are regional units described for the conservation and management of living marine resources (Sherman 1991). The Argentine Sea belongs to LME 14 of South Western Atlantic and comprises coastal environments, continental shelf, slope and ocean basins, covering 6,581,500 km^2^ of marine platform (http://www.plataformaargentina.gov.ar/en). In this area, two major marine currents coexist: the cold Malvinas and the warm Brazil currents (Boltovskoy 1979). The former, rich in nutrients, is generated from the Antarctic Circumpolar current, whereas the later moves southwards along the edge of the slope (Piola and Rivas 1997, Piola 2008). In the transition zone (from 30° to 46° S), different oceanographic processes allow a high biological production (Acha et al. 2004). From the biogeographical point of view, two zoogeographical provinces in the Argentine Sea are present. The Argentinean province extends from Cabo Frio, Brazil to Valdés peninsula, Argentina. The Magellanic province ranges from Chiloe Island, Chile, in the Pacific Ocean to the coasts of Valdés peninsula. However, in deeper waters, this biogeographical province extends further northwards to the state of Santa Catarina, Brazil (Woodward 1856, Cooke 1895, Ekman 1953, Scarabino 1977, Boschi et al. 1992, Briggs 1995, Boschi 2000a, 2000b, Spalding et al. 2007).

The Argentine coastline is more than 8,400 km in length (Venerus and Cedrola 2017). Over this large area, heterogeneous topography and variable climate can be observed. As stated by Costello et al. (2017), the oceans appear ideal for biodiversity due to unlimited water availability, large areas and less extreme temperatures respect to land. Although oceans contain more phyla and classes than land and fresh waters, only 16% of total described species are marine. Biodiversity of marine environments reaches a highest level in tropical regions, decreasing gradually towards higher latitudes (Fischer 1960, Roy et al. 1998, Engle and Summers 1999, Gray 2001, Mittelbach et al. 2007). This inverse tendency between biodiversity and latitude seems to be balanced by a higher biomass and endemism at higher latitudes (Boltovskoy et al. 2005). In the last years, some studies have been done in order to document these patterns in marine invertebrates from the South Western Atlantic (Astorga et al. 2003, Bertness et al. 2006, Diez 2006, López Gappa et al. 2006, López Gappa and Sueiro 2006, Carranza et al. 2009, Griffiths et al. 2009, Scarabino et al. 2016, Zelaya 2016, Alves et al. 2017, among others). Also, some international initiatives as NaGISA (Census of Marine Life), or SARCE (South American Research Group on Coastal Ecosystems), contribute to the knowledge of the coastal marine biodiversity.

The first zoological observations on marine biodiversity from the Argentine Sea, occurred during the 19^th^ century, when European and North American naturalists visited the South American coast (e.g. Voyage dans l’Amérique Méridionale; H.M.S. “Challenger”). These first expeditions allowed the publication of large compendiums and catalogues of marine fauna from South America (Dillwyn, 1817, Say, 1822, d´Orbigny, 1834-47, Reeve, 1843-78, E. A. Smith, 1881, 1885, among others). Subsequent local catalogues complemented these first observations with new additional data (Berg 1900, Bernasconi 1937, Carcelles 1944, Carcelles and Williamson 1951, Castellanos 1970, Escofet 1970, among others). During the second half of the 20^th^ century, several Argentine marine expeditions contributed to increase knowledge on marine invertebrate biodiversity in Argentina [e.g. R/V “*Academik Knipovich* ” (1967); R/V “*Almirante Saldanha*” (1966); R/V “*Atlantis II*”, (1971); R/V “*El Austral*” (1966-1967); R/V “*Vema*”, (1962); R/V “*Walther Herwig*” (1966-71)]. Recently (2009-2017), the R/V Puerto Deseado from the Argentinean National Research Council (CONICET) supported several field works, not only in the Argentine Sea, but also in the Antarctic Continent.

This work compiles and reviews the available information on marine invertebrate biodiversity in the Argentine Sea gathered after an exhaustive literature search.

## Taxonomic coverage

The present dataset comprises 23 phyla, 808 families, 1,662 genera and 3,064 valid species. The most represented groups are Arthropoda and Mollusca with 746 (24.35%) and 862 (28.13 %) valid species, respectively (Table 1).

**Table 1.** Number of valid species registered in WoRMS (December 2017) (worldwide distributed) and those reported in the literature for the Argentine Sea.

**Phylum**	**WoRMS**		**Argentine Sea**			
	**N° of species**	%	**N° of families**	**N° of genera**	**N° of species**	%
** Acanthocephala **	522	0.30	1	1	2	0.07
** Annelida **	13949	7.93	48	141	200	6.53
** Arthropoda **	57104	32.46	213	459	746	24.35
** Brachiopoda **	426	0.24	4	8	10	0.33
** Bryozoa **	6111	3.47	79	150	332	10.84
** Cephalorhyncha **	236	0.13	2	3	3	0.10
** Chaetognatha **	131	0.07	1	1	1	0.03
** Cnidaria **	11645	6.62	68	132	224	7.31
** Ctenophora **	200	0.11	7	7	9	0.29
** Dicyemida **	122	0.07	2	2	3	0.10
** Echinodermata **	7332	4.17	48	116	181	5.91
** Entoprocta **	190	0.11	3	3	5	0.16
** Hemichordata **	130	0.07	1	1	1	0.03
** Mollusca **	47478	26.99	206	405	862	28.13
** Nematoda **	6893	3.92	30	64	113	3.69
** Nematomorpha **	5	0.00	1	1	1	0.03
** Nemertea **	1368	0.78	6	12	30	0.98
** Phoronida **	11	0.01	1	1	2	0.07
** Platyhelminthes **	12833	7.30	33	54	75	2.45
** Porifera **	8655	4.92	49	93	250	8.16
** Rotifera **	201	0.11	1	1	1	0.03
** Sipuncula **	156	0.09	3	6	9	0.29
** Tardigrada **	209	0.12	1	1	4	0.13
**Total**	**175,907**	**100**	**808**	**1,662**	**3,064**	**100**

### Taxonomic ranks

**Phylum:** Acanthocephala

**Family:** Polymorphidae

**Genus:** *Corynosoma*

**Phylum:** Annelida

**Family**: Ampharetidae, Aphroditidae, Arenicolidae, Capitellidae, Chaetopteridae, Chrysopetalidae, Cirratulidae, Cossuridae, Dorvilleidae, Echiuridae, Eunicidae, Flabelligeridae, Glyceridae, Goniadidae, Hesionidae, Histriobdellidae, Lumbrineridae, Maldanidae, Nephtyidae, Nereidae, Nereididae, Oenonidae, Onuphidae, Opheliidae, Orbinidae, Orbiniidae, Oweniidae, Paraonidae, Pectinariidae, Pholoidae, Phyllodocidae, Pilargidae, Piscicolidae, Poecilochaetidae, Polynoidae, Sabellariidae, Sabellidae, Scalibregmatidae, Serpulidae, Sigalionidae, Spionidae, Syllidae, Terebellidae, Travisiidae, Trichobranchidae, Tubificidae, unclassified Annelida 1, Urechidae

**Genus**: *Abarenicola*, *Aglaophamus*, *Ampharete*, *Amphipolydora*, *Amphitrite*, *Anobothrus*, *Aphrodita*, *Arabella*, *Arctacama*, *Armandia*, *Artacama*, *Axiothella*, *Bathydrilus*, *Boccardia*, *Boccardiella*, *Capitella*, *Carazziella*, *Caulleriella*, *Chaetopterus*, *Cirratulus*, *Cirriformia*, *Cistenides*, *Clymenella*, *Cossura*, *Cryobdella*, *Diopatra*, *Dipolydora*, *Dispio*, *Drilonereis*, *Epigamia*, *Eteone*, *Eulalia*, *Eumida*, *Eunereis*, *Eunice*, *Eunoe*, *Euzonus*, *Exogone*, *Ficopomatus*, *Flabelligella*, *Flabelligera*, *Glycera*, *Glycinde*, *Goniada*, *Gymnonereis*, *Halosydna*, *Harmothoe*, *Hemipodia*, *Hermadion*, *Hermundura*, *Heteromastus*, *Hyalopomatus*, *Hydroides*, *Idanthyrsus*, *Kinbergonuphis*, *Laeonereis*, *Laetmonice*, *Lanice*, *Lanicides*, *Laubierpholoe*, *Leitoscoloplos*, *Levinsenia*, *Lumbrineris*, *Maldanella*, *Mammiphitime*, *Marphysa*, *Mercierella*, *Microspio*, *Nainereis*, *Neanthes*, *Neodexiospira*, *Nephtys*, *Nereis*, *Nicon*, *Ninoe*, *Notalia*, *Nothria*, *Notocirrus*, *Notomastus*, *Notopsilus*, *Onuphis*, *Ophelia*, *Ophelina*, *Ophioglycera*, *Oriopsis*, *Owenia*, *Paleanotus*, *Paralaeospira*, *Parapionosyllis*, *Paraprionospio*, *Parasabella*, *Perkinsiana*, *Petaloproctus*, *Pherusa*, *Phragmatopoma*, *Phyllochaetopterus*, *Phyllodoce*, *Phylo*, *Phynchospio*, *Pionosyllis*, *Piromis*, *Platynereis*, *Poecilochaetus*, *Polydora*, *Potamilla*, *Prionospio*, *Proceraea*, *Procerastea*, *Prochaetoparia*, *Protolaeospira*, *Romanchella*, *Sabella*, *Sabellaria*, *Salvatoria*, *Scalibregma*, *Schistomeringos*, *Scolecolepides*, *Scolelepis*, *Scoloplos*, *Serpula*, *Sigambra*, *Simplaria*, *Sphaerosyllis*, *Spio*, *Spiochaetopterus*, *Spiophanes*, *Spirorbis*, *Steggoa*, *Sthenelais*, *Stratiodrilus*, *Streblosoma*, *Syllidia*, *Syllis*, *Terebellides*, *Thalassema*, *Thelepus*, *Travisia*, *Trichobranchus*, *Typosyllis*, *Ungulites*, *Urechis*

**Phylum**: Arthropoda

**Family**: Acanthaspidiidae, Acanthephyridae, Acanthonotozomellidae, Aegidae, Aethridae, Alpheidae, Amaryllididae, Ameiridae, Ammotheidae, Ampithoidae, Ancorabolidae, Antarcturidae, Anthuridae, Aoridae, Apseudidae, Archaeobalanidae, Archaeocumatidae, Arcturidae, Aristeidae, Atelecyclidae, Austrarcturellidae, Austrobalanidae, Austrodecidae, Balanidae, Belliidae, Benthesicymidae, Blepharipodidae, Bodotriidae, Bopyridae, Branchinectidae, Bythocyprididae, Calanidae, Calappidae, Callianassidae, Callipallenidae, Campylonotidae, Cancridae, Canthocamptidae, Caprellidae, Carcinidae, Chaetiliidae, Chasmocarcinidae, Cheidae, Chthamalidae, Cirolanidae, Clausidiidae, Clausocalanidae, Cletodidae, Colomastigidae, Colossendeidae, Coronulidae, Corophiidae, Crangonidae, Cryptoniscidae, Cushmanideidae, Cyclopinidae, Cyllopodidae, Cymothoidae, Cyproideidae, Cytherideidae, Cytheruridae, Dactylopusiidae, Dendrogastridae, Desmosomatidae, Dexaminidae, Diastylidae, Diogenidae, Diosaccidae, Ectinosomatidae, Endeidae, Enteropsidae, Eophliantidae, Epialtidae, Ethusidae, Exoedicerotidae, Galenidae, Gammarellidae, Geryonidae, Gnathiidae, Grapsidae, Halacaridae, Halophilosciidae, Haploniscidae, Harpacticidae, Hemicytheridae, Hippidae, Hippolytidae, Holognathidae, Homolidae, Hyalellidae, Hyalidae, Hymenosomatidae, Hyssuridae, Idoteidae, Inachidae, Inachoididae, Iphimediidae, Ischnomesidae, Ischyroceridae, Janiridae, Joeropsididae, Lampropidae, Laophontidae, Latreilliidae, Leptanthuridae, Leptocytheridae, Leuconidae, Leucosiidae, Leucothoidae, Ligiidae, Liljeborgiidae, Limnoriidae, Lithodidae, Lophogastridae, Luciferidae, Lysianassidae, Macropipidae, Majidae, Melitidae, Miraciidae, Mithracidae, Munididae, Munnidae, Munnopsidae, Myicolidae, Mysidae, Nannastacidae, Nebaliidae, Nematocarcinidae, Neocytherideididae, Neotanaidae, Nephropidae, Nephropsidae, Normanellidae, Nymphonidae, Ochlesidae, Ocypodidae, Oedicerotidae, Oithonidae, Oplophoridae, Orthopsyllidae, Pachylasmatidae, Pachynidae, Paguridae, Palaemonidae, Pallenopsidae, Pandalidae, Panopeidae, Paracalanidae, Paradoxostomatidae, Paramunnidae, Paranthuridae, Parapaguridae, Parastenheliidae, Parthenopidae, Pasiphaeidae, Peltidiidae, Peltogastridae, Penaeidae, Peracarida, Petalophthalmidae, Photidae, Phoxocephalidae, Phoxocephalopsidae, Phoxychilidiidae, Pinnotheridae, Platyischnopidae, Platyschnopidae, Platyxanthidae, Polybiidae, Polychelidae, Pontocyprididae, Pontogeneiidae, Porcellanidae, Porcellidiidae, Portunidae, Processidae, Pseudidotheidae, Pseudotachidiidae, Rectarcturidae, Santiidae, Scalpellidae, Scyllaridae, Sebidae, Sergestidae, Serolidae, Sesarmidae, Solenoceridae, Sphaeromatidae, Squillidae, Staphylinidae, Stegocephalidae, Stenetriidae, Stenothoidae, Synopiidae, Talitridae, Tanaididae, Tegastidae, Tetrasquillidae, Thalestridae, Tisbidae, Trachyleberididae, unclassified Arthropoda 2, Upogebiidae, Uristidae, Urothoidae, Varunidae, Xanthidae, Xestoleberididae, Ydianthidae, Zobrachoidae

**Genus**: *Abyssianira*, *Acanthaspidia*, *Acanthephyra*, *Acanthocarpus*, *Acanthocyclus*, *Acantholobulus*, *Acanthonotozomoides*, *Acanthoserolis*, *Achelia*, *Achelous*, *Actaea*, *Acutiserolis*, *Advenogonium*, *Aega*, *Aegaeon*, *Aegla*, *Agauopsis*, *Allorostrata*, *Allosergestes*, *Allotanais*, *Alpheus*, *Alteutha*, *Amaryllis*, *Ambostracon*, *Ameira*, *Amonardia*, *Ampelisca*, *Amphiascoides*, *Amphiascopsis*, *Amphiascus*, *Amphibalanus*, *Ampithoe*, *Anacalliax*, *Anchistrocheles*, *Anchistylis*, *Ancinus*, *Andaniotes*, *Anoplodactylus*, *Antarctobiotus*, *Antarctomysis*, *Antarcturus*, *Antennuloniscus*, *Antennulosignum*, *Antiboreodiosaccus*, *Apohyale*, *Arcoscalpellum*, *Arenaeus*, *Argilloecia*, *Aristaeopsis*, *Armases*, *Artemesia*, *Arthromysis*, *Artystone*, *Astrurus*, *Atlantocuma*, *Atlantorchestoidea*, *Atlantoserolis*, *Atyloella*, *Atylus*, *Aurila*, *Austinixa*, *Australicythere*, *Austroaurila*, *Austrocytheridea*, *Austrodecus*, *Austrofilius*, *Austromegabalanus*, *Austronanus*, *Austropandalus*, *Austroregia*, *Balanus*, *Bathyporeiapus*, *Benthesicymus*, *Betaeus*, *Betamorpha*, *Bircenna*, *Bledius*, *Blepharipoda*, *Branchinecta*, *Brazilserolis*, *Briarosaccus*, *Bruzelia*, *Caecianiropsis*, *Caecocassidias*, *Caecognathia*, *Calanus*, *Callinectes*, *Callipallene*, *Callistocythere*, *Calyptraeotheres*, *Campylaspis*, *Campylonotus*, *Caprella*, *Carcinus*, *Cassidias*, *Cerapus*, *Ceratoserolis*, *Cetopirus*, *Chaceon*, *Chaetarcturus*, *Chasmocarcinus*, *Cheirimedon*, *Cheus*, *Chiriscus*, *Chono*, *Chorismus*, *Cilunculus*, *Cirolana*, *Claudicuma*, *Clausocalanus*, *Cleantis*, *Coenophthalmu*, *Colanthura*, *Collodes*, *Colomastix*, *Colossendeis*, *Compressoscalpellum*, *Coperonus*, *Copidognathus*, *Corystoides*, *Cristaserolis*, *Cumella*, *Cumellopsis*, *Curidia*, *Cushmanidea*, *Cyathura*, *Cyclaspis*, *Cyclopina*, *Cyllopus*, *Cymadusa*, *Cyrtograpsus*, *Cyrtoplax*, *Cytheropteron*, *Cytherura*, *Dactylopusia*, *Danielethus*, *Dardanus*, *Dendrogaster*, *Deosergestes*, *Diarthrodes*, *Diastylis*, *Disconectes*, *Dissodactylus*, *Dolichiscus*, *Drepanopus*, *Dynamenella*, *Dynoides*, *Ebalia*, *Ectinosoma*, *Edotia*, *Elminius*, *Emerita*, *Endeis*, *Enhydrosoma*, *Enhydrosomella*, *Enteropsis*, *Erikus*, *Ethusina*, *Eualus*, *Euchaetomera*, *Eudevenopus*, *Eudorella*, *Eugerdella*, *Eupelte*, *Eurycope*, *Eurypanopeus*, *Eurypodius*, *Eusergestes*, *Exhippolysmata*, *Exoediceropsis*, *Exosphaeroma*, *Fabia*, *Falklandia*, *Farfantepenaeus*, *Fissarcturus*, *Fistulobalanus*, *Fosterella*, *Frontoserolis*, *Fuegiphoxus*, *Funchalia*, *Gammaropsis*, *Gardinerosergia*, *Glyptonotus*, *Gnathia*, *Gondogeneia*, *Goodingius*, *Gracilimesus*, *Halacarellus*, *Halacarus*, *Halicarcinus*, *Haliophasma*, *Halophiloscia*, *Hansenomysis*, *Haplocheira*, *Harpacticus*, *Hemicyclops*, *Hemicythere*, *Hemicytherura*, *Hemilamprops*, *Hemingwayella*, *Henryhowella*, *Hepatus*, *Heterocythereis*, *Heterolaophonte*, *Heterosquilla*, *Hexapanopeus*, *Holostylis*, *Homola*, *Hyalella*, *Hyssura*, *Iais*, *Ianthopsis*, *Iathrippa*, *Idotea*, *Idyanthe*, *Ilyarachna*, *Iphimedia*, *Iphimediella*, *Ischyrocerus*, *Ischyromene*, *Isocladus*, *Isonebula*, *Jassa*, *Joeropsis*, *Laophonte*, *Laophontodes*, *Latreillia*, *Latreutes*, *Lebbeus*, *Lembos*, *Leptanthura*, *Leptocuma*, *Leptoserolis*, *Leptostylis*, *Leucippa*, *Leucon*, *Leucothoe*, *Leurocyclus*, *Libidoclaea*, *Libinia*, *Ligia*, *Liljeborgia*, *Limnoria*, *Linca*, *Liriopsis*, *Lissosabinea*, *Litarcturus*, *Lithodes*, *Lophogaster*, *Loxopagurus*, *Loxoreticulatum*, *Lucifer*, *Macrochiridotea*, *Macrochiridothea*, *Magellianira*, *Melita*, *Merhippolyte*, *Meridionalicythere*, *Meridiosignum*, *Mesochra*, *Mesorhoea*, *Metacarcinus*, *Metanephrops*, *Metatiron*, *Metharpinia*, *Microphoxus*, *Mixarcturus*, *Monocorophium*, *Monoculopsis*, *Moruloidea*, *Munida*, *Munna*, *Munneurycope*, *Munnogonium*, *Myropsis*, *Mysidetes*, *Mysidopsis*, *Nannocalanus*, *Natatolana*, *Nauticaris*, *Neasellus*, *Neastacilla*, *Nebalia*, *Nematocarcinus*, *Neocytherideis*, *Neohelice*, *Neojaera*, *Neolithodes*, *Neomysis*, *Neosergestes*, *Neoserolis*, *Neotanais*, *Normanella*, *Nothochthalamus*, *Notiax*, *Notobalanus*, *Notocrangon*, *Notomegabalanus*, *Notopoma*, *Nymphon*, *Oculocytheropteron*, *Oithona*, *Omonana*, *Orchestia*, *Orchomenella*, *Ornatoscalpellum*, *Orthopsyllus*, *Ostrincola*, *Ovalipes*, *Pachycheles*, *Paguristes*, *Pagurus*, *Palaemon*, *Pallenopsis*, *Pandalopsis*, *Panoppeus*, *Pantomus*, *Papillosacythere*, *Paracalanus*, *Paracymothoa*, *Paradexamine*, *Paradoxapseudes*, *Paradoxostoma*, *Parafoxiphalus*, *Paralaophonte*, *Paralomis*, *Paramonoculopsis*, *Paramphiascella*, *Paramunna*, *Paranthura*, *Parapenaeus*, *Parasergestes*, *Paraserolis*, *Parastenhelia*, *Parategastes*, *Parathalestris*, *Parawaldeckia*, *Paridotea*, *Parione*, *Pariphimedia*, *Parthenope*, *Pasiphaea*, *Patagoniella*, *Peisos*, *Pelia*, *Peltarion*, *Penaeus*, *Pentacheles*, *Perissocope*, *Perissocytheridea*, *Persephona*, *Petalidium*, *Philocheras*, *Phoxocephalopsis*, *Phoxorgia*, *Pilumnoides*, *Pilumnus*, *Pinnaxodes*, *Pinnixa*, *Planes*, *Platidotea*, *Platorchestia*, *Platyisao*, *Pleoticus*, *Pleurosignum*, *Polycheria*, *Polyonix*, *Porcellana*, *Porcellidium*, *Poti*, *Prehensilosergia*, *Probolisca*, *Probopyrus*, *Procampylaspis*, *Processa*, *Procythereis*, *Proharpinia*, *Propagurus*, *Propontocypris*, *Pseudidothea*, *Pseudione*, *Pseudiphimediella*, *Pseudobranchiomysis*, *Pseudomma*, *Pterygosquilla*, *Pyromaia*, *Quetzogonium*, *Quinquelaophonte*, *Retarcturus*, *Rhombognathus*, *Riggia*, *Robertgurneya*, *Robertsonia*, *Rochinia*, *Santia*, *Scutellidium*, *Scyllarides*, *Seba*, *Semicytherura*, *Semixestoleberis*, *Septemserolis*, *Sergestes*, *Sergia*, *Sergio*, *Serolella*, *Serolis*, *Sinelobus*, *Socarnoides*, *Sphaeroma*, *Spinolambrus*, *Stenocionops*, *Stenorhynchus*, *Stereomastis*, *Stylicletodes*, *Stylopandalus*, *Styloptocuma*, *Sursumura*, *Sympagurus*, *Synerythrops*, *Syneurycope*, *Synidotea*, *Syrrhoe*, *Systellaspis*, *Tanais*, *Tanystylum*, *Tenupedunculus*, *Tetrachaelasma*, *Tetraxanthus*, *Thymops*, *Thymopsis*, *Thysanoserolis*, *Tigriopus*, *Tiron*, *Tisbe*, *Tmetonyx*, *Tonocote*, *Triantella*, *Tryphosites*, *Tumidotheres*, *Uca*, *Ultimachelium*, *Upogebia*, *Uristes*, *Uromunna*, *Urothoe*, *Vanhoeffenura*, *Victorhensenoides*, *Waiteolana*, *Xenanthura*, *Xestoleberis*, *Xigonus*, *Xiphopenaeus*, *Xouthous*, *Zausopsis*, *Zyzzigonium*

**Phylum**: Brachipoda

**Family**: Discinidae, Frieleiidae, Terebratellidae, Terebratulidae

**Genus**: *Aneboconcha*, *Dyscritosia*, *Liothyrella*, *Magellania*, *Neorhynchia*, *Pelagodiscus*, *Syntomaria*, *Terebratella*

**Phylum**: Bryozoa

**Family**: Adeonellidae, Adeonidae, Aeteidae, Alcyonidiiade, Arachnopusiidae, Aspidostomatidae, Beaniidae, Bifaxariidae, Bitectiporidae, Bryocryptellidae, Buffonellodidae, Bugulidae, Buskiidae, Calloporidae, Calvetiidae, Calwelliidae, Candidae, Catenicellidae, Cellaridae, Cellariidae, Celleporidae, Cerioporidae, Chaperiidae, Chorizoporidae, Crepidacanthidae, Cribilinidae, Cribrilinidae, Crisiidae, Cryptosulidae, Cupuladriidae, Diaperoeciidae, Diastoporidae, Electridae, Entalophoridae, Escharinidae, Exochellidae, Farciminariidae, Farrellidae, Favoelariidae, Flustridae, Fredericellidae, Frondiporidae, Gigantoporidae, Haywardozoontidae, Hippoporidridae, Hippothoidae, Horneridae, Immergentiidae, Inversiulidae, Lacernidae, Lekythoporidae, Lichenoporidae, Lyroporidae, Membraniporidae, Microporellidae, Microporidae, Myriaporidae, Odmoneidae, Oncousoeciidae, Onichocellidae, Orbituliporidae, Phidoloporidae, Philodoporidae, Plagioeciidae, Porinidae, Pseudidmoneidae, Pustuloporidae, Romancheinidae, Romncheinidae, Schizoporellidae, Sclerodomidae, Scrupariidae, Smittinidae, Stomatoporidae, Tubuliporidae, Umbonulidae, unclassified Bryozoa 1, Vesicularidae, Walkeriidae

**Genus**: *Adeonella*, *Adeonellopsis*, *Aetea*, *Aimulosia*, *Alcyonidium*, *Alderina*, *Alloeoflustra*, *Amastigia*, *Amathia*, *Amphiblestrum*, *Andreella*, *Antarctothoa*, *Apiophragma*, *Arachnopusia*, *Aspericreta*, *Aspidostoma*, *Austroflustra*, *Beania*, *Bicrisia*, *Bientalophora*, *Bowerbankia*, *Buffonellodes*, *Bugula*, *Bugulina*, *Buskia*, *Caberea*, *Callopora*, *Calloporina*, *Calvetia*, *Camptoplites*, *Canda*, *Carbasea*, *Catadysis*, *Cellaria*, *Cellarinella*, *Celleporella*, *Celleporina*, *Chaperia*, *Chaperiopsis*, *Chartella*, *Chiastosella*, *Chondriovelum*, *Chorizopora*, *Codonellina*, *Columnella*, *Conopeum*, *Cookinella*, *Cornucopina*, *Crepidacantha*, *Crisia*, *Crisularia*, *Cryptostomaria*, *Cryptosula*, *Dartevellia*, *Diaperoecia*, *Discoporella*, *Disporella*, *Domosclerus*, *Electra*, *Ellisina*, *Escharina*, *Escharoides*, *Euginoma*, *Eurystrotos*, *Exochella*, *Farrella*, *Fasciculipora*, *Favostimosia*, *Fenestrulina*, *Figularia*, *Filisparsa*, *Flustrapora*, *Foveolaria*, *Galeopsis*, *Gigantopora*, *Gregarinidra*, *Haywardozoon*, *Hemismittoidea*, *Himantozoum*, *Hippadenella*, *Hippomonavella*, *Hippoporina*, *Hippothoa*, *Hornera*, *Ichthyaria*, *Idmidronea*, *Idmonea*, *Immergentia*, *Inversiula*, *Jolietina*, *Kenoaplousina*, *Lacerna*, *Lageneschara*, *Lichenopora*, *Mecynoecia*, *Melicerita*, *Membranicellaria*, *Membranipora*, *Menipea*, *Metroperiella*, *Micropora*, *Microporella*, *Monastesia*, *Myriapora*, *Neoflustra*, *Neothoa*, *Nevianipora*, *Notoplites*, *Odontoporella*, *Ogivalia*, *Orthoporidra*, *Orthoporidroides*, *Osthimosia*, *Paracellaria*, *Parafigularia*, *Parasmittina*, *Phonicosia*, *Plagioecia*, *Platonea*, *Platychelyna*, *Plesiothoa*, *Porella*, *Pseudidmonea*, *Retepora*, *Reteporella*, *Reteporellina*, *Romancheina*, *Salicornaria*, *Sclerodomus*, *Scruparia*, *Scrupocaberea*, *Scrupocellaria*, *Securiflustra*, *Sertella*, *Smittina*, *Smittoidea*, *Sphaerulobryozoon*, *Spiroporina*, *Stephanollona*, *Stomatopora*, *Stomhypselosaria*, *Talivittaticella*, *Tricellaria*, *Tubulipora*, *Turbicellepora*, *Turritigera*, *Umbonula*, *Villicharixa*, *Walkeria*, *Xylochotridens*

**Phylum**: Cephalorhyncha

**Family**: Echinoderidae, Priapulidae.

**Genus**: *Echinoderes*, *Priapulopsis*, *Priapulus*

**Phylum**: Chaetognatha

**Family**: Sagittidae

**Genus**: *Sagitta*

**Phylum**: Cnidaria

**Family**: Acontiophoridae, Actiniidae, Actinostolidae, Aglaopheniidae, Alcyoniidae, Andvakiidae, Anthoptilidae, Bathyphelliidae, Blackfordiidae, Boloceroididae, Bougainvilliidae, Campanulariidae, Campanulinidae, Caryophylliidae, Clavulariidae, Corallimorphidae, Corymorphidae, Corynidae, Cyaneidae, Diadumenidae, Drymonematidae, Edwardsiidae, Epizoanthidae, Eudendriidae, Flabellidae, Halcampidae, Haleciidae, Haliplanellidae, Halipteridae, Haloclavidae, Halopteridae, Halopterididae, Hebellidae, Hormathiidae, Hydractiniidae, Hydridae, Isanthidae, Isididae, Isophellidae, Kirchenpaueriidae, Lafoeidae, Limnactiniidae, Lovenellidae, Lychnorhizidae, Metridiidae, Mitrocomidae, Niobiidae, Oceaniidae, Olindiidae, Pelagiidae, Pennatulidae, Periphyllidae, Phialellidae, Plumulariidae, Primnoidae, Renillidae, Rhodaliidae, Sagartiidae, Sertulariidae, Stomolophidae, Stylasteridae, Syntheciidae, Tetraplatidae, Thyroscyphidae, Tiarannidae, Tubulariidae, Ulmaridae, unclassified Cnidaria 1

**Genus**: *Abietinella*, *Acryptolaria*, *Actinauge*, *Actinostola*, *Actinothoe*, *Aglaophenia*, *Alcyonium*, *Amphianthus*, *Amphisbetia*, *Andvakia*, *Anemonia*, *Antholoba*, *Anthoptilum*, *Anthothoe*, *Armadillogorgia*, *Artemidactis*, *Atolla*, *Aulactinia*, *Aurelia*, *Austroneophellia*, *Billardia*, *Bimeria*, *Blackfordia*, *Bolocera*, *Boloceroides*, *Botryon*, *Bougainvillia*, *Boungainvillia*, *Bunodactis*, *Calliactis*, *Calycella*, *Campanularia*, *Caryophyllia*, *Chrysaora*, *Clytia*, *Corymorpha*, *Corynactis*, *Coryne*, *Desmonema*, *Diadumene*, *Drymonema*, *Dynamena*, *Echinisis*, *Ectopleura*, *Epiactis*, *Epizoanthus*, *Eucheilota*, *Eudendrium*, *Filellum*, *Flabellum*, *Glandulactis*, *Gonothyraea*, *Grammaria*, *Halecium*, *Halipteris*, *Halisiphonia*, *Halopteris*, *Harenactis*, *Hartlaubella*, *Hebella*, *Hormathia*, *Hybocodon*, *Hydra*, *Hydractinia*, *Hydrodendron*, *Inferiolabiata*, *Isoparactis*, *Isophellia*, *Isosicyonis*, *Isotealia*, *Kirchenpaueria*, *Lafoea*, *Limnactinia*, *Lychnorhiza*, *Lytocarpia*, *Mitrocomella*, *Monactis*, *Monastaechas*, *Nauthisoe*, *Nemertesia*, *Niobia*, *Obelia*, *Olindias*, *Orthopyxis*, *Oulactis*, *Parabunodactis*, *Parahalcampa*, *Paraisometridium*, *Paranthus*, *Paraphelliactis*, *Parascyphus*, *Parathuiaria*, *Pariactis*, *Peachia*, *Pennatula*, *Periphylla*, *Phacellophora*, *Phelliactis*, *Phelliogeton*, *Phialella*, *Phlyctenanthus*, *Phymactis*, *Plumarella*, *Plumularia*, *Pseudoparactis*, *Ramirezia*, *Renilla*, *Rhizogeton*, *Rhodalia*, *Rhodelinda*, *Sagartianthus*, *Sarsia*, *Schizotricha*, *Scolanthus*, *Sertularella*, *Sicyonis*, *Silicularia*, *Sporadopora*, *Stauroteca*, *Staurotheca*, *Stegella*, *Stegopoma*, *Stomolophus*, *Stygiomedusa*, *Stylaster*, *Symplectoscyphus*, *Synthecium*, *Tetraplatia*, *Tricnidactis*, *Urticina*, *Urticinopsis*, *Zoanthina*

**Phylum**: Ctenophora

**Family**: Atollidae, Beroidae, Cestidae, Lampeidae, Lyroctenidae, Mertensiidae, Pleurobrachiidae

**Genus**: *Beroe*, *Callianira*, *Cestum*, *Lampea*, *Lyrocteis*, *Mnemiopsis*, *Pleurobrachia*

**Phylum**: Dicyemida

**Family**: Conocyemidae, Dicyemidae

**Genus**: *Conocyema*, *Dicyema*

**Phylum**: Echinodermata

**Family**: Abertellidae, Aeropsidae, Amphilepididae, Amphiuridae, Antedonidae, Arbaciidae, Asteriidae, Asterinidae, Asterostomatidae, Astropectinidae, Benthopectinidae, Chiridotidae, Cidaridae, Ctenocidaridae, Ctenodiscidae, Cucumariidae, Echinasteridae, Echinidae, Elpidiidae, Ganeriidae, Goniasteridae, Goniopectinidae, Gorgonocephalidae, Heliasteridae, Korethrasteridae, Laetmogonidae, Luidiidae, Mellitidae, Odontasteridae, Ophiacanthidae, Ophiactidae, Ophiodermatidae, Ophiolepididae, Ophiomyxidae, Ophiuridae, Parechinidae, Phyllophoridae, Poraniidae, Prenasteridae, Pseudachasteridae, Psolidae, Pterasteridae, Schizasteridae, Solasteridae, Stichasteridae, Synallactidae, Temnopleuridae, Urechinidae

**Genus**: *Abatus*, *Abertella*, *Aceste*, *Achlyonice*, *Acodontaster*, *Allostichaster*, *Amphilepis*, *Amphiodia*, *Amphiophiura*, *Amphipholis*, *Amphipodia*, *Amphiura*, *Anasterias*, *Anteliaster*, *Arbacia*, *Asterina*, *Astrochlamys*, *Astrohamma*, *Astropecten*, *Astrotoma*, *Athyonidium*, *Austrocidaris*, *Bathybiaster*, *Bathyplotes*, *Brisaster*, *Calyptraster*, *Ceramaster*, *Cheiraster*, *Chiridota*, *Cladaster*, *Cladodactyla*, *Cosmasterias*, *Ctenodiscus*, *Cycethra*, *Delopatagus*, *Diplasterias*, *Diplodontias*, *Diplopteraster*, *Echinaster*, *Elpidia*, *Encope*, *Florometria*, *Ganeria*, *Glabraster*, *Gorgonocephalus*, *Hemioedema*, *Hemipholis*, *Henricia*, *Hippasteria*, *Hymenaster*, *Isometra*, *Labidiaster*, *Laetmogone*, *Leptychaster*, *Lethasterias*, *Lophaster*, *Loxechinus*, *Luidia*, *Luidiaster*, *Mediaster*, *Molpadiodemas*, *Neomilaster*, *Notocidaris*, *Odontaster*, *Ophiacantha*, *Ophiactis*, *Ophiocamax*, *Ophioceres*, *Ophiochondrus*, *Ophiocten*, *Ophiogona*, *Ophiolebella*, *Ophioleuce*, *Ophiolimna*, *Ophiolycus*, *Ophiomastus*, *Ophiomitrella*, *Ophiomusium*, *Ophiomyxa*, *Ophionotus*, *Ophioperla*, *Ophioplinthus*, *Ophioplocus*, *Ophiosparte*, *Ophiosteira*, *Ophiozonella*, *Ophiura*, *Pentactella*, *Pentamera*, *Peribolaster*, *Perissasterias*, *Perknaster*, *Porianopsis*, *Promachocrinus*, *Psalidaster*, *Pseudarchaster*, *Pseudechinus*, *Pseudocnus*, *Pseudostichopus*, *Psilaster*, *Psolidium*, *Psolus*, *Pteraster*, *Remaster*, *Scotoplanes*, *Sigmodota*, *Smilasterias*, *Solaster*, *Staurocucumis*, *Sterechinus*, *Taeniogyrus*, *Trachythyone*, *Tremaster*, *Tripylaster*, *Tripylus*, *Urechinus*

**Phylum**: Entoprocta

**Family**: Barentsiidae, Loxosomatidae, Pedicellinidae

**Genus**: *Barentsia*, *Loxosomella*, *Pedicellina*

**Phylum**: Hemichordata

**Family**: Rhabdopleuridae

**Genus**: Rhabdopleura

**Phylum**: Mollusca

**Family**: Acmaeidae, Acteocinidae, Acteonidae, Aeolidiidae, Anatomidae, Anomiidae, Aplustridae, Argonautidae, Astartidae, Barleeiidae, Bathydorididae, Bathyspinulidae, Borsoniidae, Buccinidae, Cadlinidae, Caecidae, Calliostomatidae, Callochitonidae, Calyptraeidae, Cancellariidae, Capulidae, Cardiidae, Carditidae, Cassidae, Cavoliniidae, Cerithiidae, Cetoconchidae, Chaetopleuridae, Chitonidae, Chromodorididae, Cingulopsidae, Cliidae, Clionidae, Cocculinidae, Cochlespiridae, Cochliopidae, Collonidae, Columbellidae, Condylocardiidae, Conidae, Corambidae, Corbulidae, Crassatellidae, Cuspidariidae, Cuvierinidae, Cyamiidae, Cyclochlamyidae,Cylichnidae, Cymbuliidae, Dentaliidae, Diaphanidae, Discodorididae, Donacidae, Dorididae, Dotidae, Drillidae, Drilliidae, Eatoniellidae, Eatonielllidae, Ellobiidae, Entalinidae, Enteroctopodidae, Epitoniidae, Eubranchidae, Eulimellinae, Eulimidae, Facelinidae, Fasciolariidae, Fissurellidae, Flabellinidae, Gadilidae, Gaimardiidae, Galeommatidae, Gastrochaenidae, Gonatidae, Goniodoridae, Goniodorididae, Hemiarthridae, Hermaeidae, Hiatellidae, Ischnochitonidae, Kellielidae, Lametilidae, Laonidae, Lasaeidae, Laternulidae, Lepetidae, Leptochitonidae, Limacinidae, Limapontiidae, Limidae, Limifossoridae, Limifossoridae, Limopsidae, Liotiidae, Littorinidae, Loliginidae, Lologinidae, Lottiidae, Lucinidae, Lyonsiellidae, Lyonsiidae, Mactridae, Malletiidae, Mangeliidae, Margaritidae, Marginellidae, Mathildidae, Mesodesmatidae, Montacutidae, Mopaliidae, Muricidae, Myidae, Mytilidae, Mytillidae, Nacellidae, Nassariidae, Naticidae, Neilonellidae, Neoleptonidae, Neomeniidae, Newtoniellidae, Notaeolidiidae, Nuculanidae, Nuculidae, Nystiellidae, Ocotpodidae, Octopodidae, Octopoidae, Olivellidae, Olividae, Omalogyridae, Ommastrephidae, Onchidorididae, Onychoteuthidae, Orbitestellidae, Ostreidae, Pandoridae, Pectinidae, Pendromidae, Peraclidae, Periplomatidae, Pharidae, Philinidae, Philobryidae, Pholadidae, Plakobranchidae, Planorbidae, Pleurobranchaeidae, Pleurobranchiidae, Plicatulidae, Pnemodermatidae, Polyceridae, Poromyidae, Propeamussiidae, Protocuspidariidae, Pseudomelatomidae, Pteriidae, Pulsellidae, Pyramidellidae, Pyroteuthidae, Ranellidae, Raphitomidae, Retusidae, Rhabdidae, Rissoidae, Sareptidae, Scissurellidae, Seguenziidae, Seguenzioidae, Semelidae, Siliculidae, Simrothiellidae, Siphonariidae, Skeneidae, Solariellidae, Solecurtidae, Solemyidae, Solenidae, Spiolidae, Tegulidae, Tellinidae, Terebridae, Teredinidae, Tergipedidae, Thraciidae, Thyasiridae, Tindariidae, Tofanellidae, Tonnidae, Tritoniidae, Trochidae, Turbinidae, Turritelidae, Ungulinidae, Vanikoridae, Velutinidae, Veneridae, Vesicomyidae, Volutidae, Volutomitridae, Wemersoniollidae, Yoldiidae

**Genus**: *Abra*, *Acanthina*, *Acanthodoris*, *Acanthopleura*, *Acesta*, *Acharax*, *Acmaea*, *Acteocina*, *Acteon*, *Adamussium*, *Adelomelon*, *Adipicola*, *Admete*, *Adontorhina*, *Adrana*, *Aeolidia*, *Aequipecten*, *Aesopus*, *Aforia*, *Agladrillia*, *Alia*, *Aloidis*, *Altenaeum*, *Alvania*, *Amarilladesma*, *Amauropsis*, *Amiantis*, *Amphissa*, *Anachis*, *Anatoma*, *Ancula*, *Angulus*, *Anomacme*, *Anomalocardia*, *Antistreptus*, *Aplysiopsis*, *Argeneuthria*, *Argentovoluta*, *Argobuccinum*, *Argonauta*, *Aspalima*, *Astarte*, *Asthenothaerus*, *Astyris*, *Atomiscala*, *Aulacomya*, *Austrochlamys*, *Austrocominella*, *Axinulus*, *Bankia*, *Barleeia*, *Bathydoris*, *Bathyspinula*, *Belalora*, *Bentheledone*, *Berghia*, *Berthella*, *Bostrycapulus*, *Brachidontes*, *Brachiodontes*, *Brevinucula*, *Brookula*, *Buccinanops*, *Cadlina*, *Cadulus*, *Caecum*, *Calliostoma*, *Callochiton*, *Capulus*, *Cardiomya*, *Carditamera*, *Carditella*, *Carditopsis*, *Carolesia*, *Catillopecten*, *Cavinetnea*, *Cavolinia*, *Cerithiella*, *Cerodrillia*, *Cetoconcha*, *Chaetopleura*, *Chlamys*, *Chrysallida*, *Clio*, *Clione*, *Cocculina*, *Conchoceles*, *Conus*, *Coralliophila*, *Corambe*, *Corbula*, *Coroniscala*, *Coronium*, *Crassinella*, *Crenella*, *Crepidula*, *Crepipatella*, *Cuspidaria*, *Cuthona*, *Cuvierina*, *Cyamiocardium*, *Cyamiun*, *Cyclocardia*, *Cyclochlamys*, *Cyclopecten*, *Cyclostrema*, *Cylichna*, *Cymbulia*, *Dacrydium*, *Dallocardia*, *Darina*, *Delectopecten*, *Dentalium*, *Dermatomya*, *Diaphana*, *Diaulula*, *Diodora*, *Diplodonta*, *Donax*, *Doris*, *Doryteuthis*, *Doto*, *Drillia*, *Duplicaria*, *Eatoniella*, *Eledone*, *Elysia*, *Emiliostraca*, *Ennucula*, *Ensis*, *Enteroctopus*, *Entodesma*, *Epicodakia*, *Epitonium*, *Ercolania*, *Eubranchus*, *Eulimastoma*, *Eulimella*, *Eulimostraca*, *Eumetula*, *Eurhomalea*, *Euspira*, *Eutivela*, *Falsilunatia*, *Falsimargarita*, *Falsitromina*, *Fictonoba*, *Fissidentalium*, *Fissurela*, *Fissurella*, *Fissurellidea*, *Flabellina*, *Flexopecten*, *Fuegotrophon*, *Fusitriton*, *Gaimardia*, *Gargamella*, *Geitodoris*, *Genaxinus*, *Glypteuthria*, *Gonatus*, *Graneledone*, *Halistylus*, *Harpovoluta*, *Haurakia*, *Hebetancylus*, *Heleobia*, *Hemiarthrum*, *Hemiliostraca*, *Hiatella*, *Holoplocamus*, *Homalopoma*, *Illex*, *Iothia*, *Ischnochiton*, *Jaspidella*, *Jukesena*, *Kellia*, *Kelliella*, *Kerguelenatica*, *Kidderia*, *Kurtiella*, *Laevilitorina*, *Lamellaria*, *Laona*, *Lasaea*, *Laternula*, *Laubiericoncha*, *Ledella*, *Lepidopleurus*, *Leptochiton*, *Leucosyrinx*, *Leukoma*, *Limacina*, *Limatula*, *Limea*, *Limifossor*, *Limopsis*, *Linucula*, *Lissarca*, *Lissotesta*, *Lithophaga*, *Littoridina*, *Lodderia*, *Loligo*, *Loripes*, *Lucapinella*, *Lucinoma*, *Luzonia*, *Lyonsia*, *Lyonsiella*, *Lyrodus*, *Macoma*, *Macromphalina*, *Mactra*, *Magallana*, *Malletia*, *Malvinasia*, *Mangelia*, *Margarella*, *Margarites*, *Marseniopsis*, *Martialia*, *Mathilda*, *Melanella*, *Mendicula*, *Meteuthria*, *Minicymbiola*, *Miomelon*, *Mitrella*, *Moroteuthis*, *Mulinia*, *Munditia*, *Muricopsis*, *Musculus*, *Muusoctopus*, *Muussoctopus*, *Mya*, *Myonera*, *Mysella*, *Mytilimeria*, *Mytilus*, *Nacella*, *Natica*, *Neilonella*, *Neobuccinum*, *Neolepton*, *Neomenia*, *Nettastoma*, *Newnesia*, *Notaeolidia*, *Notocochlis*, *Nucula*, *Nuculana*, *Nuttallochiton*, *Nuttalochiton*, *Octopus*, *Odontocymbiola*, *Odostomia*, *Oenopota*, *Okenia*, *Olivancillaria*, *Olivella*, *Omalogyra*, *Onoba*, *Onychoteuthis*, *Orbitestella*, *Ostrea*, *Pagodula*, *Pandora*, *Panopea*, *Papuliscala*, *Parabuccinum*, *Paradmete*, *Paraeuthria*, *Parathyasira*, *Pareuthria*, *Parficulina*, *Parmaphorella*, *Parvanachis*, *Parvaplustrum*, *Parviturbo*, *Patelloida*, *Pellilitorina*, *Pelseneeria*, *Peltodoris*, *Pendroma*, *Peracle*, *Periploma*, *Pertusiconcha*, *Perumytilus*, *Petricola*, *Phidiana*, *Philine*, *Philobrya*, *Phlyctiderma*, *Photinastoma*, *Photinula*, *Pisolamia*, *Pitar*, *Plawenia*, *Plaxiphora*, *Pleurobranchaea*, *Pleurotomella*, *Plicatula*, *Pododesmus*, *Policordia*, *Polycera*, *Polyschides*, *Pontiothauma*, *Poromya*, *Powellisetia*, *Prelametila*, *Prisogaster*, *Pristigloma*, *Probuccinum*, *Prodoris*, *Propebela*, *Propeleda*, *Prosipho*, *Protocuspidaria*, *Provocator*, *Prunum*, *Pseudokellia*, *Pteria*, *Pterigioteuthis*, *Pulsellum*, *Puncturella*, *Pupatonia*, *Pusillina*, *Pyrene*, *Pyrunculus*, *Raeta*, *Rapana*, *Retrotapes*, *Retusa*, *Rhabdus*, *Rhinoclama*, *Robsonella*, *Rocellaria*, *Rostanga*, *Savatieria*, *Scissurella*, *Scurria*, *Scutopus*, *Seguenzia*, *Semele*, *Semicassis*, *Semimytilus*, *Semirossia*, *Silicula*, *Sinezona*, *Sinuber*, *Siphonaria*, *Siphonodentalium*, *Skenella*, *Solariela*, *Solen*, *Sphenia*, *Spirotropis*, *Spongiobranchaea*, *Strigilla*, *Strombiformis*, *Tagelus*, *Tawera*, *Tectonatica*, *Tegula*, *Tellina*, *Terebra*, *Teredo*, *Thecacera*, *Thesbia*, *Thielea*, *Thracia*, *Thyasira*, *Tindaria*, *Toledonia*, *Tonicia*, *Tonna*, *Tractolira*, *Transempitar*, *Trenchia*, *Tritonia*, *Trochita*, *Tromina*, *Trophon*, *Trophonopsis*, *Tropidomya*, *Turbonilla*, *Turritella*, *Turritellopsis*, *Typhlodaphne*, *Tyrinna*, *Vesicomya*, *Volutomitra*, *Volvarina*, *Waldo*, *Wemersoniella*, *Xymenopsis*, *Yoldia*, *Yoldiella*, *Zeadmete*, *Zidona*, *Zygochlamys*

**Phylum**: Nematoda

**Family**: Acuariidae, Anisakidae, Anoplostomatidae, Anticomidae, Axonolaimidae, Camacolaimidae, Chromadoridae, Comesomatidae, Desmodoridae, Diplopeltidae, Draconematidae, Enchelidiidae, Enoplidae, Ethmolaimidae, Haliplectidae, Leptolaimidae, Leptosomatidae, Linhomoeidae, Microlaimidae, Monhysteridae, Monoposthiidae, Oncholaimidae, Phanodermatidae, Selachinematidae, Siphonolaimoidea, Sphaerolaimidae, Thoracostomopsidae, Tripyloididae, unclassified Nematoda 1, Xyalidae

**Genus**: *Anoplostoma*, *Anticoma*, *Aponema*, *Araeolaimus*, *Bathylaimus*, *Camacolaimus*, *Cantracaecum*, *Cervonema*, *Chromadora*, *Chromadorita*, *Comesoma*, *Contracaecum*, *Cosmocephalus*, *Crestanema*, *Daptonema*, *Deontostoma*, *Desmodora*, *Desmolaimus*, *Didelta*, *Diplolaimelloides*, *Draconema*, *Enoplus*, *Euchromadora*, *Eumorpholaimus*, *Eurystomina*, *Fenestrolaimus*, *Graphonema*, *Halichoanolaimus*, *Haliplectus*, *Hopperia*, *Laimella*, *Leptolaimus*, *Linhystera*, *Metalinhomoeus*, *Metoncholaimus*, *Microlaimus*, *Monhystera*, *Monoposthia*, *Neochromadora*, *Nudora*, *Odontophora*, *Oncholaimellus*, *Oncholaimus*, *Paraethmolaimus*, *Paralinhomoeus*, *Paramesacanthion*, *Paramonohystera*, *Parasaveljevia*, *Perspiria*, *Phanoderma*, *Pontonema*, *Prochromadora*, *Pseudocella*, *Pseudosteineria*, *Ptycholaimellus*, *Sabatieria*, *Siphonolaimus*, *Sphaerolaimus*, *Steineridora*, *Terschellingia*, *Theristus*, *Thoracostoma*, *Tripyloides*, *Viscosia*

**Phylum**: Nematomorpha

**Family**: Nectonematidae

**Genus**: *Nectonema*

**Phylum**: Nemertea

**Family**: Amphiporidae, Lineidae, Malacobdellidae, Panorhynchidae, Tetrastemmatidae, Valenciniidae

**Genus**: *Amphiporus*, *Baseodiscus*, *Cerebratulus*, *Gastropion*, *Huilkia*, *Lineus*, *Malacobdella*, *Panorhynchus*, *Parapolia*, *Parborlasia*, *Tetrastemma*, *Wiotkenia*

**Phylum**: Phoronida

**Family**: unclassified Phoronida

**Genus**: *Phoronis*

**Phylum**: Platyhelminthes

**Family**: Bdellouridae, Bothriocephalidae, Bucephalidae, Capsalidae, Cathetocephalidae, Diclidophoridae, Echeneibothriidae, Echinobothriidae, Echinostomatidae, Eutetrarhynchidae, Fecampiidae, Gyrocotylidae, Hemiuridae, Hexabothriidae, Lacistorhynchidae, Macrovalvitrematidae, Mazocraeidae, Meidiamidae, Microphallidae, Onchobothriidae, Opecoelidae, Paraberrapecidae, Phyllobothriidae, Plagiostomidae, Pterobothriidae, Rhinebothriidae, Sphyriocephalidae, Strigeidae, Taxa incertae sedis, Tentaculariidae, Tetrabothriidae, Triaenophoridae, Umagillidae.

**Genus**: *Acanthobothrium*, *Anonchocephalus*, *Anthobothrium*, *Bothriocephalus*, *Bucephalus*, *Calliobothrium*, *Callitetrarhynchus*, *Callorhynchocotyle*, *Cardiocephaloides*, *Cathetocephalus*, *Clestobothrium*, *Collastoma*, *Coronocestus*, *Crossobothrium*, *Dasyrhynchus*, *Diclidophora*, *Dollfusiella*, *Echinostoma*, *Fecampia*, *Grillotia*, *Guidus*, *Gyrocotyle*, *Halysioncum*, *Hepatoxylon*, *Heteronybelinia*, *Kronborgia*, *Lacistorhynchus*, *Lecithochirium*, *Levinseniella*, *Macruricotyle*, *Maritrema*, *Mazocraes*, *Mecistobothrium*, *Meidiama*, *Microphallus*, *Neogrubea*, *Neomacrovalvitrema*, *Neopterinotrematoides*, *Nicolasia*, *Notomegarhynchus*, *Opecoeloides*, *Orygmatobothrium*, *Paraberrapex*, *Parachristianella*, *Parahemiurus*, *Plagiostomun*, *Prosorhynchoides*, *Pseudanthocotyloides*, *Pterobothrium*, *Rhinebothrium*, *Symcallio*, *Synsiphonium*, *Tetrabothrius*, *Tetrasepta*

**Phylum**: Porifera

**Family**: Acarnidae, Ancorinidae, Axinellidae, Baeriidae, Biemnidae, Callyspongiidae, Chalinidae, Clionaidae, Coelosphaeridae, Darwinellidae, Dendoricellidae, Dictyonellidae, Dysideidae, Esperiopsidae, Geodiidae, Grantiidae, Guitarridae, Halichondriidae, Halisarcidae, Hamacanthidae, Hyalonematidae, Hymedesmiidae, Isodictyidae, Latrunculiidae, Latrunculina, Leucaltidae, Leucascidae, Leucosoleniidae, Microcionidae, Mycalidae, Myxillidae, Niphatidae, Petrosiidae, Phellodermidae, Phloeodictyidae, Plakinidae, Polymastiidae, Raspailiidae, Rossellidae, Spongiidae, Spongillidae, Stelligeridae, Stylocordylidae, Suberitidae, Sycettidae, Tedaniidae, Tethyidae, Tetillidae, Thorectidae

**Genus**: *Amphilectus*, *Amphimedon*, *Artemisina*, *Auletta*, *Axinella*, *Biemna*, *Callyspongia*, *Calyx*, *Caulophacus*, *Chalinula*, *Cinachyra*, *Ciocalypta*, *Clathria*, *Cliona*, *Dasychalina*, *Dendrilla*, *Dictyonella*, *Dragmacidon*, *Dysidea*, *Echinoclathria*, *Ephydatia*, *Esperiopsis*, *Eurypon*, *Fibula*, *Fibulia*, *Gellius*, *Geodia*, *Grantia*, *Guitarra*, *Halichondria*, *Haliclona*, *Haliclonissa*, *Halicnemia*, *Halisarca*, *Hamacantha*, *Hemigellius*, *Hyalonema*, *Hymedesmia*, *Hymenancora*, *Hymeniacidon*, *Hyrtios*, *Inflatella*, *Iophon*, *Isodictya*, *Latrunculia*, *Leucandra*, *Leucascus*, *Leucettusa*, *Leuconia*, *Leucosolenia*, *Lissodendoryx*, *Megaciella*, *Microxina*, *Mycale*, *Myxilla*, *Neopetrosia*, *Oceanapia*, *Pachychalina*, *Pachychalina*, *Petrosia*, *Phakellia*, *Phelloderma*, *Phorbas*, *Pione*, *Plakina*, *Plicatellopsis*, *Polymastia*, *Pseudosuberites*, *Pyloderma*, *Radiospongilla*, *Raspailia*, *Rhizaxinella*, *Rossella*, *Scalarispongia*, *Scopalina*, *Semisuberites*, *Spongia*, *Spongosorites*, *Stelletta*, *Stelodoryx*, *Stylocordyla*, *Suberites*, *Sycon*, *Tedania*, *Tentorium*, *Tethya*, *Tethyopsis*, *Tetilla*, *Topsentia*, *Trochospongilla*, *Ulosa*, *Volzia*

**Phylum**: Rotifera

**Family**: Philodinidae

**Genus**: *Anomopus*

**Phylum**: Sipuncula

**Family**: Golfingiidae, Phascolionidae, Themistidae

**Genus**: *Golfingia*, *Nephasoma*, *Nephastoma*, *Onchnesoma*, *Phascolion*, *Themiste*

**Phylum**: Tardigrada

**Family**: Batillipedidae

**Genus**: *Batillipes*

## Methods

**Spatial coverage**: The spatial coverage of this project ranged from 35°51'16.98'S/ 55°40'20.27'W to 55°11'27.81'S/ 66°7'6.21'W. It comprises coastal environments, the continental shelf and slope, and ocean basins (Argentine Marine Platform).

**Literature survey and quality control description**: A comprehensive literature review was carried out. It included scientific publications, technical reports, and uploaded data to OBIS database during the NaGISA (Census of Marine Life) and SARCE projects. The reviewed literature allowed the compilation of marine invertebrate taxa reported by the Argentine Sea.The taxonomic status of the taxa were contrasted with updated literature, and corroborated using World Register of Marine Species databases (WoRMS 2017). Thus, the number of phyla, families, genera, and current valid species combinations are reported. However, no taxonomic revisions of the cited species were undertaken. These results provide an updated checklist of marine invertebrate knowledge on the Argentine Sea. For each phylum, the percentage of valid species living in the Argentine Sea was compared with the global percentage reported by WoRMS (http://www.marinespecies.org/aphia.php?p=stats). This analysis allowed us to assess the status of knowledge for each phylum in a global and regional context.

**Data resources.** The dataset herein reported has been revised and updated from a published dataset as part of a larger project through OBIS, as a result of the Census of Marine Life-NaGISA project [Marine Invertebrate from Argentina, Uruguay and Chile. v1.4. ArOBIS Centro Nacional Patagónico. Dataset/Occurrence. http://arobis.cenpat-conicet.gob.ar:8081/resource?r=arobis-marineinvertebrate].

**Figure 1.**
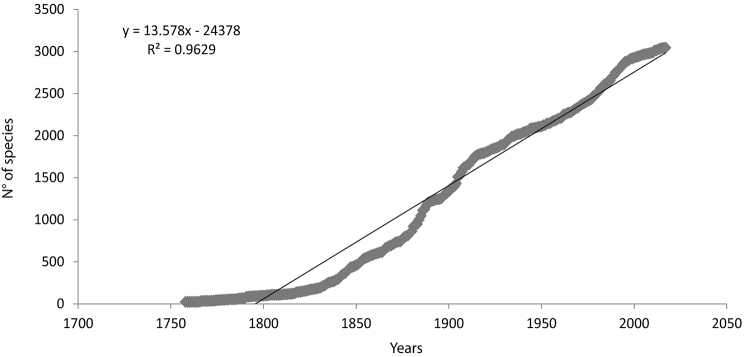
Cumulative curve for valid marine invertebrate species reported as living in the Argentine Sea (South Western Atlantic). Each dot in the figure represents the year when the taxa was described (and subsequently reported in the literature as living in the Argentine Sea).

**Data Analysis**: A cumulative species analysis was carried out to estimate the status of knowledge of marine invertebrate biodiversity of Argentine Sea. This study was done by using the Clench model (v2 = (a*v1)/(1+(b*v1)), applied by Jimenez-Valverde and Hortal (2003). In this work, we defined as effort units the number of species described per year from 1758 to 2017. In this analysis, only the valid species were considered. Each dot in Figure 1 represents the year when the valid species was described (and subsequently reported in the literature as living in the Argentine Sea). The number of described valid species per year in the region was tested using the Statistica 5.1 program, with the Simplex & Quasi-Newton adjust model. In case of no data fitting the Clench model, another one would be used.

**Object name**: Darwin Core Archive Marine Invertebrate from Argentina, Uruguay and Chile (in part).

**Character encoding**: UTF-8

**Format name**: Darwin Core Archive format.

**Format version**: 1.0

**Distribution**: http://arobis.cenpat-conicet.gob.ar:8081/resource?r=arobis-marineinvertebrate

**Publication date of data**: 2016-11-17

**Language**: English

**Licenses of use**: The publisher and rights holder of this work is ArOBIS Centro Nacional Patagónico. This work is licensed under a Creative Commons Attribution Non Commercial (CC-BY-NC) 4.0 License.

**Metadata language**: English

**Date of metadata creation**: 2015-09-07

**Hierarchy level**: Dataset

## Discussion

The large surface of the Argentinean Marine Platform and Coasts, together with the low number of valid reported species of marine invertebrates, denote that more research is required to increase the knowledge of this group in the South Western Atlantic Ocean and particularly, in the Argentine Sea. The data here compiled did not fit to the Clench model (y= (a*x)/1+b*x)). The obtained curve was y = ((6.33037*x)/ (1+ ((-0.00198)*x)); R=0.98121. As the value of b is almost zero, the obtained curve could be considered as linear. When fitting the data to a linear curve, the formula was y= 13.578x – 24378 (R² = 0.9629). This could be attributable to the fact that species mentioned in the literature for the Argentine Sea would be less than 50% of the expected marine invertebrate species present in the region (Fig. 1).

**Figure 2.**
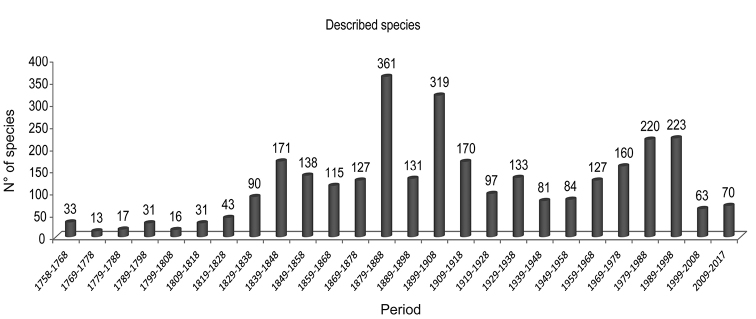
Number of valid marine invertebrate species described per decade that were subsequently mentioned in the literature as living in the Argentine Sea.

During the last two centuries, an average of twelve species had been described per year as living in the Argentine Sea. At the beginning of the 19^th^ century, the descriptions were completely based on material collected by European and North American expeditions (Fig. 2). The creation of the Museo Argentino de Ciencias Naturales (MACN) in 1812 contributed to increase the knowledge and descriptions of marine invertebrates (Penchaszadeh 2012). By the end of the 19^th^ century and the beginning of 20^th^ two “golden periods” were observed (1879-1888 and 1899-1908). During these two periods the amount of described species was considerably increased probably associated to global marine expeditions. One of them was undoubtedly the “Challenger Expedition” of 1873-76, which described more that 4,000 new species over the world. The reports of this expedition are considered as one of the greatest progresses in the knowledge of the world´s natural history. By the end of the 20^th^ century, another pulse, of almost 450 species, was newly described for Argentine waters, in the period 1979-1998 (Fig. 2). This fact could be probably associated to the consolidation of specialists in taxonomy in Argentina and the return of scientists exiled during the military dictatorship (1976-1983). During these 20 years (1979–1998) 30 % of the Nematoda, Bryozoa and Brachiopoda registered in Argentine waters were described. However, the phyla Mollusca and Arthropoda were still the most represented groups during that period. Finally, in the last years (beginning of 21^st^ century), new species are being described, mainly promoted by the scientific system of Argentina (MINCyT, CONICET), international projects (Census of Marine Life) and open access databases (OBIS, WoRMS). Nonetheless, the knowledge of marine invertebrate biodiversity is still low in the region.

The Kingdom Animalia comprises 29 invertebrate phyla (WoRMS), however, only six phyla have not been recorded as living in the Argentine Sea (Table 1). These are Cycliophora, Gastrotricha, Gnathostomulida, Orthonectida, Placozoa and Xenacoelomorpha. The phylum Arthropoda and Mollusca constitute around 50 % of the reported marine invertebrates. However, the percentage of Argentinean marine Arthropoda is lower compared to the global knowledge, revealing that this group is far to be resolved in the region. In contrast to that, the mollusks percentage is more consistent. Some groups as Bryozoa, Cnidaria, Porifera and Echinodermata exceed the global registered percentage reported by WoRMS (2017). The observed percentage of the phylum Nemertea coincides with the worldwide registered in WoRMS. Nevertheless, only 30 species have been reported as living in the southwestern Atlantic, suggesting that the number of known nemertean is still low. In addition, 70% of Nemertea species was described in the Northern Hemisphere (Kajihara et al. 2008). This could indicate that new Argentinean nemerteans could be described in the future. Research focused on marine invertebrate biodiversity in Argentina is currently growing. Additionally, some young researchers on invertebrate taxonomy are being trained towards a scientific career. On the other hand, the financial support provided by the government is still scarce.

**Figure 3.**
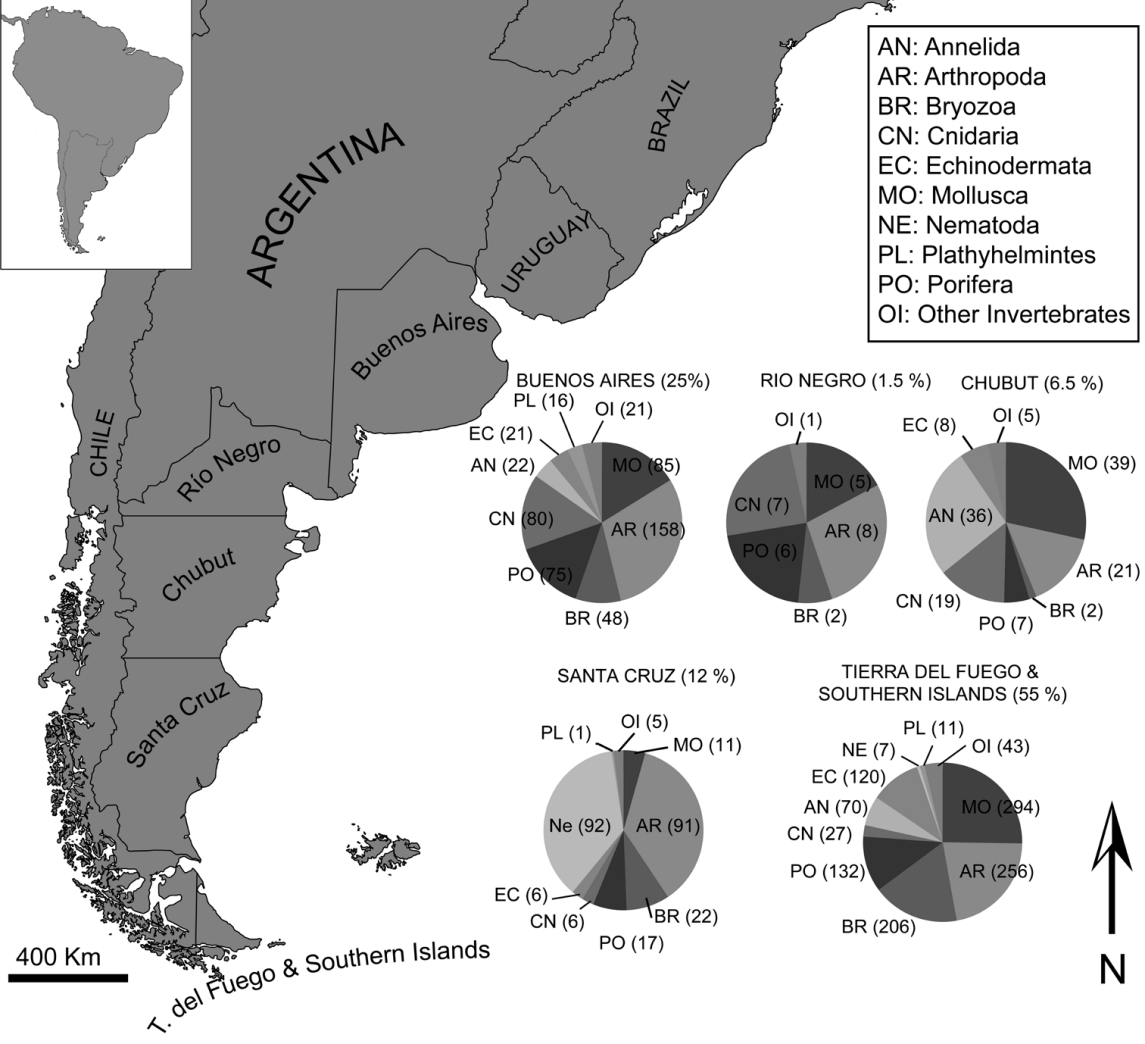
Distribution of main taxonomic marine invertebrate groups. The parenthesys after the province indicates the percentage of species mentioned as living in each province. The parenthesys after the phyllum initials indicates the number of species mentioned in the literature.

A distribution analysis of the species is a complex issue, due to, in several cases, the literature examined named “Argentine Sea” or “Argentine Coast” as a locality. This is the case of 955 records of species cited for the Argentine Sea without a precise locality. However, distribution patterns by provinces were made excluding those 955 records and estimating the percentage for the main taxonomic groups in order to elucidate hot spots in the Argentine Sea (Fig. 3). It is clear that the Magellan region is the most studied region of the Argentine Sea with 1166 (55%) mentioned species in the literature followed by the Buenos Aires province coast with 526 (25 %). Few records were exclusively mentioned for the Río Negro Province in the literature; only 29 (1,5 %) species were named for this area. Santa Cruz and Chubut provinces, with 251 (12 %) and 137 (6,5 %) reported species respectively, present more species than Río Negro but the number of reported species is still low compared to Tierra del Fuego and Buenos Aires provinces. In general terms, the phylum Mollusca and Arthropoda were the most mentioned groups along the Argentine Sea. Nevertheless, the phylum Nematoda in the Santa Cruz province and Annelida (mostly Polychaeta) in Chubut, were widely studied (Fig. 3). The fact that more species are described in the southern region of the Argentine Sea could be attributable to the concentration of oceanographic campaigns that were performed by international initiatives when travel to Antartica or passing from Pacific to Atlantic Ocean (around Tierra del Fuego and Southern Islands). The major biodiversity encountered in the southern tip of the Southwest Atlantic also could be attributable to an inverse biodiversity pattern that was previously registerd in Southwest Atlantic higher latitudes for some intertidal rocky shore invertebrates (Palomo et al 2011) or other taxa as asellote isopods (Doti et al. 2014). The increasing in biodiversity in high latitudes could also be attributable to the presence of high extentions of hard bottoms that permit the settlement of invertebrates and the fact that most Magellanic species that occur in southern Chile extend to the Southwest Atlantic (López Gappa et al. 2006).

In Argentina, the main factors that modify benthic communities are habitat degradation and disturbance, urban development, dredging and resuspension of sediment, establishment of ports, tourism-associated impact, global and local aquatic contamination sources, and fisheries (Bigatti and Penchaszadeh 2008). Notably, bottom trawling dominates coastal and deep-sea fishing in the Argentine platform. This fishery produces a large number of discards of benthic invertebrates, accounting up to 80 % of the catch (Orensanz et al. 2008). In order to provide an adequate management of the natural resources, studies on coastal management, conservation and distribution patterns have been carried out (Sullivan and Bustamante 1999, Barragán et al. 2003, Cusson and Bourget 2005, Cañete et al. 2008, Miloslavich et al. 2011, among others).

Finally, biological invasions of different organisms (algae, mollusks, hydroids, bryozoans, ascidiaceans and crustaceans) have negatively affected local marine biodiversity, as well as, regional economy (Orensanz et al. 2002, Penchaszadeh et al. 2005, Bigatti and Penchaszadeh, 2008, Schwindt 2008). A total of 28 marine exotic species and 43 cryptic species have been reported as living in the Argentine Sea (Orensanz et al. 2002), while the number is increasing in the last years. The impact of biological invasions constitutes a serious problem to marine invertebrate biodiversity in Argentine Sea and consequently affects descriptions of new species, even before of their description. The results of this checklist suggest the importance of studies focused on marine invertebrate biodiversity in the southern tip of South America, where some hot spots, as the Protected Marine Area Burdwood bank, harbor great abundance and diversity of endemic species (Miloslavich et al 2011). New studies on marine invertebrate biodiversity will provide consistent data for the generation of management policies tending to create new marine protected areas and the conservation of the species´habitats.

## Supplementary materials

Supplementary material 1 List of Valid species reported as living in the Argentine Sea Data type: occurence This is an open access article distributed under the terms of the Creative Commons Attribution License (CC BY 4.0), which permits unrestricted use, distribution, and reproduction in any medium, provided the original author and source are credited.Gregorio Bigatti, Javier Signorelli
